# Feasibility and Measurement Quality of Home‐Based Smartphone Spirometry in Children With Suspected Asthma: A Multicentre, Prospective Study

**DOI:** 10.1002/ppul.71768

**Published:** 2026-08-02

**Authors:** Antonia Knopek, Hanna Fabinyi, Viktoria Zach, Anna Beliveau, Nina Luisa Hoekstra, Carlotta Nüssing, Katharina Kainz, Swantje Weisser, Anna Zschocke, Angela Zacharasiewicz, Christiane Lex

**Affiliations:** ^1^ Department of Pediatrics and Adolescent Medicine, Division of Pediatric Cardiology and Intensive Care Medicine University Hospital Goettingen Goettingen Germany; ^2^ Department of Paediatrics and Adolescent Medicine Klinik Ottakring Vienna Austria; ^3^ Department of Pediatric and Adolescent Medicine, Pediatrics III Medical University Innsbruck Austria

**Keywords:** asthma diagnosis, mHealth, pediatrics, pulmonary function tests, spirometry quality control, telemedicine

## Abstract

**Background:**

Diagnosing asthma in children is challenging due to the episodic nature of symptoms and limitations of in‐clinic lung function testing. Smartphone spirometry offers a potential solution by enabling repeated measurements in real‐life settings. However, data on its feasibility in children with suspected but unconfirmed asthma are limited.

**Objective:**

To evaluate compliance, measurement quality, and agreement between manual and automated grading of smartphone spirometry in children with suspected asthma.

**Methods:**

In this multicentre, prospective study, 102 children aged 5–16 years with suspected asthma received a smartphone spirometer and were instructed to perform daily spirometry for 7 days. Measurement quality was assessed using ATS/ERS 2019 criteria by both manual review and automated device grading. Associations with age, supervision, and social factors were analysed.

**Results:**

Of 101 children with technically valid data, 93.1% performed at least one measurement series at home, while 31.7% completed measurements on all seven study days. Clinically usable measurement quality (grades A–C for both FEV_1_ and FVC) was achieved at least once by 61.7% (manual grading) and 74.5% (automated grading). Automated grading showed strong correlation and high agreement with manual grading (ICC = 0.90), but slightly overestimated quality. Measurement quality was not significantly associated with age, supervision, or social factors.

**Conclusion:**

Smartphone spirometry is feasible for home‐based lung function assessment in children with suspected asthma. Compliance and measurement quality were moderate but acceptable for clinical interpretation in most participants. Automated grading aligns well with manual review though potential discrepancies should be considered in clinical decision‐making.

## 1 Introduction

The diagnostic evaluation of asthma in children and adolescents remains a clinical challenge. The process typically relies on symptom history, clinical findings, and lung function testing. However, the episodic and variable nature of asthma symptoms in children often leads to in‐clinic lung function assessments during asymptomatic intervals. This can result in non‐pathological findings even in children with asthma. Therefore, objective findings can be absent, despite being essential for establishing a reliable diagnosis. This diagnostic gap may contribute to overdiagnosis as well as missed diagnoses [1]. To address this diagnostic uncertainty, particularly in cases with fluctuating symptoms, one promising approach is the use of smartphone‐based spirometry in everyday settings.

Smartphone spirometers are portable devices that connect to a smartphone or tablet via Bluetooth and enable repeated lung function testing outside clinical environments. Various models from different manufacturers have been evaluated in studies involving both adults and children [2, 3, 4]. These studies have demonstrated the general feasibility and accuracy of smartphone spirometry in paediatric populations with asthma.

However, existing studies have primarily been focused on patients with an established asthma diagnosis, who are therefore typically already receiving anti‐inflammatory therapy and have prior experience with lung function testing. These populations are not representative of children in whom asthma is suspected but not yet confirmed. In such cases, the reliability and quality of home‐based spirometry remain insufficiently studied.

In particular, there is a need to assess clinical usability of smartphone spirometry for diagnostic purposes in children with suspected asthma. Open questions include adherence to measurement protocols in a home setting, the acceptability and reproducibility of test attempts without professional supervision, and the validity of automated grading algorithms for measurement quality embedded in the device software. To date, no studies have directly compared the automated interpretation of spirometry quality of the device with expert manual grading.

As primary outcome this study therefore aimed to assess the feasibility of home‐based smartphone‐spirometry in children with suspected asthma over 1 week. Feasibility was conceptualised as an overarching construct encompassing two complementary dimensions: Compliance with the measurement protocol and measurement quality, composed of acceptability and reproducibility. As secondary outcomes, agreement between manual and automated grading and the exploration of factors potentially associated with measurement compliance and quality were defined.

## 2 Methods

A multicentre study was conducted at three study centers— Goettingen (Universitätsmedizin Göttingen, Germany), Vienna (Klinik Ottakring (Wiener Gesundheitsverbund), Austria) and Innsbruck (Universitätskinderklinik Innsbruck, Austria)—from October 2022 to May 2025. The study protocol was approved by the respective ethics committees (Approval numbers: 20/6/22 (Goettingen), EK 22‐136‐0622 (Vienna), 1023/2023 (Innsbruck)). Written informed consent was obtained from all parents and children following the provision of age‐appropriate study information materials. This research forms part of a non‐randomised prospective observational study titled “ESUBAK” (Evaluation of Smartphone‐Spirometry and Body‐Plethysmography in Diagnosing Asthma in Children).

### 2.1 Participants and Study Design

Children aged 5–16 years who presented with suspected asthma (recurrent cough, wheezing and dyspnoea) to the paediatric pulmonology outpatient clinic were screened for eligibility and included in the study accordingly. If an asthma diagnosis could not be confirmed during the initial appointment according to the European Respiratory Society clinical practice guidelines [5] using only spirometry with bronchodilation, participants were provided with a smartphone spirometer for further evaluation of asthma. Participants were excluded if they met any of the following criteria: (a) contraindications to performing spirometry, such as acute dyspnoea, a history of spirometry‐induced bronchospasm, or acute contagious infections (b) severe comorbidities, such as complex congenital heart disease or oncological diseases (c) inability to adequately perform spirometry, e.g. due to severe cognitive impairment (d) use of inhaled corticosteroids within the 6 weeks prior to inclusion (e) absence of a mobile device compatible with the smartphone spirometer.

### 2.2 Smartphone Spirometry

Lung function measurements were performed at home over seven consecutive days using the AioCare smartphone spirometer by Healthup S.A. (Warsaw, Poland). This portable device pairs via Bluetooth with an app installable on Android or iOS smartphones and tablets (AioCare Patient/AioCare Doctor; versions used: 2.13.1‐3.0.16). Both the device and the associated app were explained to the families. Additionally, the measurement procedure was practiced under the supervision of a Medical Technical Specialist in Functional Diagnostics, who also provided an overview of how to recognise good measurement quality. After initial training in the office setting, lung function measurements were performed at home without further professional supervision, providing real‐world data. The aim was to perform daily measurements for 1 week, repeated measurements were encouraged, especially if symptoms appeared. There was no set limit for a maximum amount of measurement series. The measurements were automatically uploaded to an online platform (AioCare Panel), where they were reviewed after the device was returned. The following data were visible on the platform for each performed examination: date and time, spirometric measurement data including FVC, FEV_1_, FEV_1_/FVC (value, lower‐limit‐of‐normal (LLN), z‐score, % predicted, percentile), flow‐volume‐ and volume‐time‐curve and a grading of measurement quality. A measurement attempt refers to a single forced expiratory manoeuvre. Up to eight individual attempts are grouped into a measurement series. The term measurement is used as an overarching term encompassing one or more measurement series performed within a session. The app provided basic automated feedback on measurement quality. Colour‐coded indicators displayed whether each manoeuvre met ATS/ERS criteria, and standardised messages alerted participants if a measurement series was incomplete or technically invalid. The families independently decided whether parental supervision was needed during the measurements. This was documented in a questionnaire completed after the measurements were concluded. Additionally, the ‘International Study of Asthma and Allergies in Childhood’ (ISAAC) questionnaire [6] (Phase III Core Questionnaire, German translation) was provided to assess the impact of various social factors. From the available variables parental education, number of siblings and household smoking were selected based on their potential relevance to the study's outcomes. Other ISAAC variables were not included in the investigation of possible confounders because no direct association with compliance or measurement quality was anticipated.

### 2.3 Measurement Quality

Measurement quality was graded according to the ATS/ERS 2019 guideline for standardisation of spirometry manually and compared to automated grading by the application software, which was also based on these criteria [7]. Manual grading was performed by a single reviewer across all study centres following an initial calibration phase with a second reviewer to ensure consistent application of the criteria. In detail, all measurement series for both FEV_1_ and FVC were graded A to F by assessing acceptability and reproducibility. Reproducibility is defined as the difference between the largest and second‐largest FEV_1_ or FVC value within a measurement series. The criteria for acceptability include, among others, the absence of relevant artefacts and the performance of a good forced expiration without glottic closure, which reaches the end of forced expiration (EOFE). For grade A, representing the best measurement quality, three acceptable measurements with a difference of ≤ 150 mL had to be recorded for both FEV_1_ and FVC in children over 6 years of age. Clinical usability was defined as grades A–C in accordance with Perrem et al. [8]. Manual grading served as the primary outcome for feasibility assessment, while automated grading was treated as a secondary outcome to assess agreement with manual grading.

### 2.4 Data Analysis

Feasibility was evaluated descriptively across its two dimensions: compliance and measurement quality. Fulfilment of the study protocol was assessed based on completion of at least one measurement series. Clinically usable quality was defined as grades A‐C for both FEV_1_ and FVC in at least one measurement series. No specific feasibility thresholds were predefined, and findings were interpreted in the context of existing literature. Descriptive analyses were performed using medians and interquartile ranges (IQR) for non‐normally distributed continuous variables and relative frequencies for categorical variables. Spearman's correlation was used to assess associations between ordinal or continuous variables. Group comparisons were conducted using the Mann–Whitney test or Kruskal–Wallis test, as appropriate. To quantify absolute agreement beyond linear correlation between manual and automated grading, a two‐way mixed‐effects intraclass correlation coefficient (ICC, absolute agreement, single measures) was calculated. Exploratory partial correlation analyses were conducted controlling for potential confounders. A significance level of *p* < 0.05 was considered statistically significant. Missing data were not imputed; analyses were performed using available data only. Participants without technically valid spirometry measurements were excluded from analyses requiring lung function data; for questionnaire‐based variables, analyses were conducted based on available responses. All statistical analyses were performed in SPSS Statistics (version 29; IBM Corporation).

## 3 Results

**Figure 1:**
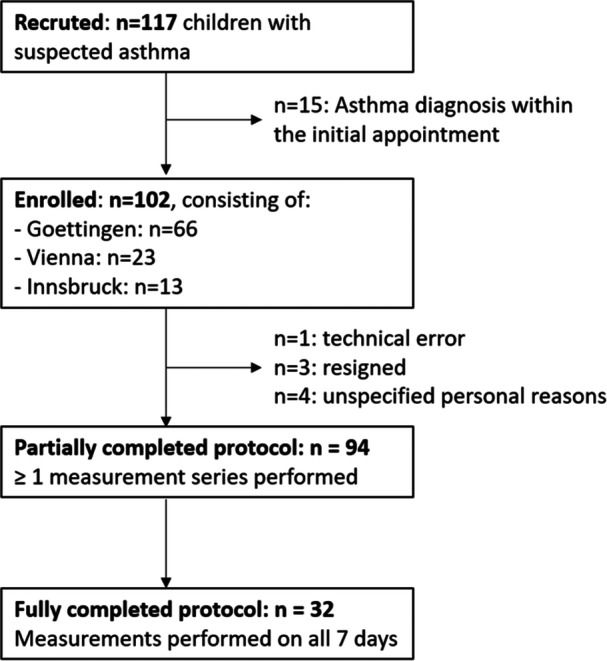
Participant selection.

A total of 117 children were recruited for the study. In 15 cases, a diagnosis of asthma was established during the initial appointment in the clinic. The remaining 102 children were included in this study and received a smartphone spirometer. Three children discontinued participation during the measurement period but retained consent for data use; their available data were included in the analyses. The completion of the study protocol for these children is depicted in Figure 1, and their baseline characteristics are in Table 1.

**Table 1:** Baseline characteristics at date of enrolment.

	Participants (*n* = 102)
Age, median (IQR)	10 (8–13)
Sex	
– Male, *n* (%)	53 (52.0%)
– Female, *n* (%)	49 (48.0%)
Weight, kg, median (IQR)	37.6 (29.1–50.0)
Height, m, median (IQR)	143 (131–160)
Body mass index, median (IQR)	18.1 (16.0–20.4)
Lung function parameters (smartphone spirometer)^a^	
– FEV_1_: z‐Score, median (IQR) % predicted, median (IQR)	−0.26 (−1.01–0.93) 97 (88‐111)
– FVC: z‐Score, median (IQR) % predicted, median (IQR)	0.07 (−0.81‐0.87) 101 (90‐110)
– FEV_1_/FVC: z‐Score, median (IQR) % predicted, median (IQR)	−0.14 (−0.95‐0.50) 99 (94‐103)

^a^ for *n* = 88 participants with available results from the initial clinic visit.

### 3.1 Primary Outcome

#### 3.1.1 Compliance

94/101 children (93.1%) with technically valid data performed at least one measurement series at home within 1 week. 32/101 (31.7%) children conducted measurements on all 7 days and therefore fully completed the study protocol. The 101 children performed a median of 7 measurement series (IQR 4–8) on 6 days (IQR 4–7) within 1 week. In total, 649 measurement series were performed across the three study centres, comprising 2271 individual attempts.

#### 3.1.2 Grading of Measurement Quality

**Figure 2:**
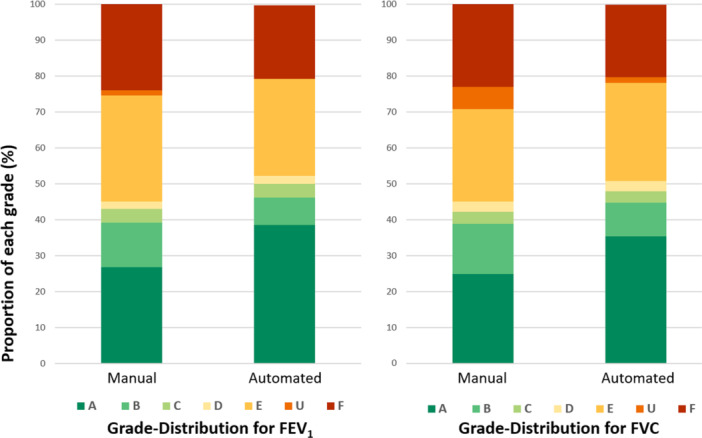
Distribution of measurement grades for FEV_1_ and FVC (in %). [Color figure can be viewed at wileyonlinelibrary.com]

In the manual assessment, FEV_1_ was deemed acceptable in 1,049 measurement attempts (46.2%) and FVC in 1009 attempts (44.4%). In comparison, the automated assessment classified 1219 attempts (53.7%) as acceptable for FEV_1_ and 1184 attempts (52.1%) for FVC. The combination of acceptability and reproducibility thresholds resulted in the distribution of quality grades shown in Table 2 and Figure 2.

**Table 2:** Grading of measurement quality—distribution of grades.

	A–C	D‐E	U	F
FEV1				
– Manual grading, *n* (%)	280 (43.1%)	205 (31.6%)	1 (0.2%)	163 (25.1%)
– Automated grading, *n* (%)	325 (50.1%)	190 (29.3%)	0	134 (20.6%)
FVC				
– Manual grading, *n* (%)	274 (25.0%)	185 (28.5%)	41 (6.3%)	149 (23.0%)
– Automated grading, *n* (%)	312 (48.1%)	196 (30.2%)	10 (1.5%)	131 (20.2%)

Of the 649 measurement series, 280 (43.1%) were graded as clinically usable (grade A–C) for FEV_1_ and 274 (42.2%) for FVC in the manual assessment. In comparison, the automated grading classified 325 series (50.1%) as grade A–C for FEV_1_ and 312 (48.1%) for FVC. In total, 242 of the 649 series (37.3%) were rated A–C for both FEV_1_ and FVC in the manual assessment, resulting in a clinically usable FEV_1_/FVC ratio. In the automated assessment, this applied to 285 series (43.9%). In 82/649 (12.6%) measurement series, a discrepancy between automated and manual grading resulted in a difference in clinical usability; in all such cases, the difference in grading exceeded a single quality grade. Specifically, 64 series were classified as clinically usable only by the automated approach, whereas the opposite occurred in 18 cases. Spearman correlation analysis showed a positive association between the number of measurement series with grade A–C for both FEV1 and FVC and the total number of measurement series performed by a child (manual assessment: *R* = 0.404, *p* < 0.001, automated assessment: *R* = 0.493, *p* < 0.001). Among the 94 participants who completed at least one measurement series, 58 children (61.7%) achieved at least one session graded A–C for both FEV_1_ and FVC in the manual assessment, compared to 70 children (74.5%) in the automated assessment—a difference of 12.8%. The median number of such sessions per child was 2 in both manual (IQR 0–4) and automated grading (IQR 0–5).

### 3.2 Secondary Outcomes

#### 3.2.1 Agreement between Manual and Automated Grading

When comparing the number of measurements graded A–C per child, the automated grading algorithm showed a strong linear correlation with manual assessments (*r* = 0.916, *p* < 0.001). The intraclass correlation coefficient (ICC) for single measures was 0.903 (95% CI [0.845, 0.938], *p* < 0.001), indicating a high level of agreement. On average, the automated method classified more measurement series per patient as clinically usable compared to the manual method (mean difference = 0.46, SD = 1.38).

#### 3.2.2 Agreement Between Clinic and Home Setting

Agreement in achieving at least one measurement series graded A–C was assessed between the supervised in‐clinic smartphone spirometry at the initial visit and home measurements for 90 children with data available from both settings. Using manual grading, 27/90 children (30%; automated grading: 46 (51.1%)) achieved grade A–C in both settings and 23/90 (25.6%; automated: 12 (13.3%)) in neither setting. Nine children (10%; automated: 10 (11.1%)) achieved grade A–C in clinic but not at home, whereas 31 children (34.4%; automated: 22 (24.4%)) did so at home despite not achieving it in clinic. McNemar tests indicated a significant difference between settings (manual: *p* < 0.001; automated: *p* = 0.050), while individual‐level agreement was low (manual: Cohen's *κ* = 0.16, *p* = 0.088; automated: *κ* = 0.19, *p* = 0.062).

### 3.3 Associated Factors

#### 3.3.1 Age

Spearman correlation analyses showed no significant association between age and any measurement‐related parameter, including the number of measurement days or series, the number of acceptable FEV_1_/FVC attempts, or the number of high‐quality measurements for both manual and automatic grading (Supporting Information S1: Table S1). In addition, Mann–Whitney U tests revealed no significant difference in age distribution between children who achieved at least one measurement graded A–C for both FEV_1_ and FVC in the same session and those who did not, either in automated grading (*U* = 817.5, *Z* = −0.196, *p* = 0.845, *r* = −0.02) or manual grading (*U* = 999.5, *Z* = −0.162, *p* = 0.871, *r* = −0.02).

#### 3.3.2 Supervision During the Measurements at Home

For 77/94 children (81.9%), who completed ≥ 1 measurement series at home, supervision during those measurements was documented. 23/77 children (29.9%) completed their measurements independently, 43 (55.8%) were supervised and for 11 children (14.3%) supervision varied. Among the supervised children 35/43 (81.4%) were always supervised by the same person, while 8/43 (18.6%) were supervised by different people.

**Figure 3:**
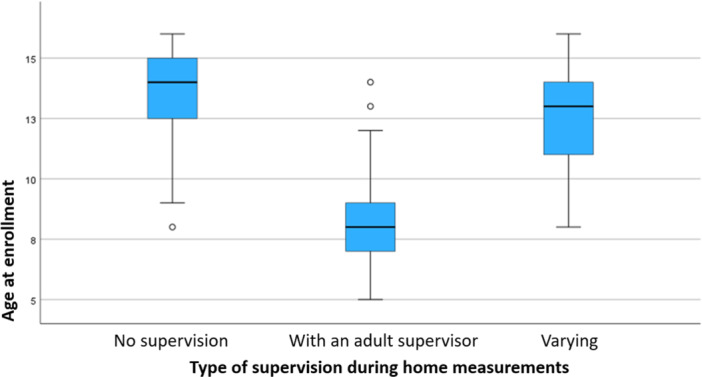
Age distribution across supervision groups during home spirometry measurements. [Color figure can be viewed at wileyonlinelibrary.com]

A Kruskal–Wallis test revealed a significant association between age and supervision status (H(2) = 42.87, *p* < 0.001, *η* ^2^= 0.56, *n* = 77) as shown in Figure 3, with median ages of 14 years (no supervision), 8 years (supervision), and 13 years (varying supervision). Post‐hoc pairwise comparisons with Bonferroni correction revealed that supervised children were significantly younger than those measuring independently (*p* < 0.001) or with varying supervision (*p* < 0.001), while the latter two groups did not differ significantly from each other (*p* = 1.0).

Spearman correlation analyses showed no significant relationship between supervision and measurement‐related parameters such as measurement quality (Supporting Information S1: Table S1).

#### 3.3.3 Social Factors

98/102 parents (96,1%) filled out the ISAAC questionnaire; however, not all parents answered every question. The children had a median of 1 sibling (IQR 1‐2). 46/94 children (48.9%) had at least one parent with higher academic education. Eighty‐two parents answered the questions regarding their smoking habits. In 30/82 (36.6%) cases, there was at least one smoking household member, and in 7/82 cases (8.5%) smoking took place inside the child's home.

Social factors (parental education, number of siblings, household smoking) showed limited association with measurement compliance or quality in Spearman analysis (Supporting Information S1: Table S1). Weak negative correlations were observed between the number of siblings and the number of measurement days (*r* = −0.208, *p* = 0.048) and measurement series (*r* = −0.23, *p* = 0.028). Partial correlation analyses controlling for age confirmed these associations for measurement days (rs = −0.230, *p* = 0.029, *df *= 88) and series (rs = −0.231, *p* = 0.029, *df *= 88), while they confirmed no significant associations between social factors and markers of measurement quality (all rs ≤ 0.148, all ps ≥ 0.228, *df* = 66–73).

## 4 Discussion

To our knowledge, this is the first study to investigate measurement quality and its associated factors for home‐based smartphone spirometry in children with suspected but unconfirmed asthma. This is a subgroup characterised by variable disease expression and limited prior experience with spirometric measurements, which poses specific challenges for achieving acceptable manoeuvres. The participants used an AioCare smartphone‐spirometer over a consecutive 7‐day period at home. The principal findings of this study are as follows: first, compliance with the measurements was moderate; second, approximately 40% of measurement series were of clinically usable measurement quality, although two thirds of children were able to produce measurements of clinically usable quality; and third, automated grading by the device showed high agreement with manual grading.

To determine the extent to which the children adhered to the study protocol, we quantified compliance based on the number and distribution of completed spirometry attempts. While almost all children performed at least one measurement series at home, only one‐third of participants conducted measurements on all seven designated study days. This highlights the challenge of sustaining repeated lung function testing over time outside a clinical setting and aligns with a previous study, which found that teenagers with asthma completed only 44% of the requested weekly spirometry measurements using a mobile spirometer [2]. In a different study, young adults with asthma demonstrated a much higher rate of completed measurements [9], indicating that young age could be a limiting factor. It is known that in children with asthma, reduced compliance leads to a significant decline in valid home‐spirometry‐data over time [10]. For PEF‐monitoring, this effect has even been demonstrated over a short time period [11]. Further investigation into factors contributing to low compliance rates—such as technical difficulties, lack of motivation or time constraints—is needed to improve participation rates in future studies.

Measurement quality was evaluated according to ATS/ERS grading criteria, focusing on the proportion of measurement series with clinically usable quality grades (A–C). For FEV_1_ as well as FVC, less than half of the measurement series reached those grades in manual and automated grading. This is consistent with previous studies in paediatric home spirometry showing that over 50% of measurement series failed to meet ATS/ERS standards without professional supervision [12, 13]. In comparison, in a study with 1624 children (mean age: 12.3 years), who performed measurements in clinic with a traditional spirometer, 85% of measurements reached grades A‐C for FEV_1_ and 72,4% for FVC [8].

A possible reason for the low number of clinically usable quality grades in home spirometries may be a significant proportion of sessions graded E, partly due to only a single measurement attempt being performed. This has been noted in previous studies [12, 13] and was also observed in our data. Other possible reasons, that should be further studied, include the effort‐dependence required to meet end‐of‐forced‐expiration (EOFE) criteria and the fact that the ATS/ERS standards provide adjusted reproducibility thresholds only for children younger than 7 years. In 2022, Perrem et al. proposed a possible alternative algorithm for measurement quality grading in children, which used smaller ranges of expired volumes to rate repeatability [8]. Although this approach is not currently included in the official ATS/ERS guideline, it could help improve grading accuracy in younger children and might be considered as an additional alternative in future studies on measurement quality in children.

Around two‐thirds of the children in our cohort who performed at least one measurement series achieved grades A‐C for both FEV_1_ and FVC at least once, thereby enabling interpretation of the FEV_1_/FVC ratio. This supports the potential clinical value of smartphone spirometry for diagnostic assessment when multiple attempts are conducted over a longer period. In a study with adults with asthma using a smartphone spirometer over 21 days, an even higher rate was achieved, with 96% of participants receiving grade A once or more [4]. This difference could be age‐dependent.

We observed a positive correlation between the total number of measurement series and the number of clinically usable measurement series (*p* < 0.001), suggesting that perseverance and practice improve test quality. This could indicate a learning effect, which should be further studied and might be an important consideration for clinical application and patient education.

The automated quality grading algorithm embedded in the spirometry device demonstrated a strong correlation with manual expert assessment and a high intraclass correlation coefficient (ICC = 0.90). However, the device tended to slightly overestimate quality compared to manual grading. This discrepancy could lead to an overoptimistic impression of measurement reliability if automated grading is used as the sole quality control. The discordances between automated and manual grading were not limited to borderline quality thresholds. Therefore, manual review or improved automated algorithms may be necessary in clinical or research settings to ensure accurate quality assessment. The possible clinical implications of these discrepancies, particularly in the context of asthma diagnosis and monitoring, should be further explored. Perhaps in the future, the use of artificial intelligence could help improve automated quality grading.

The comparison of measurement quality between supervised in‐clinic at the initial visit and home‐based smartphone spirometry revealed a setting effect rather than stable, person‐specific performance limitations. Difficulties in achieving acceptable measurements were not confined to the same children across settings, as reflected by the low individual‐level agreement (manual grading: *κ* = 0.16). The slightly higher success rate at home may reflect repeated practice over several days, whereas the in‐clinic assessment represented a first‐time use of the device at the end of a long study visit.

In our cohort, age did not significantly affect measurement quality or grading differences. This contrasts with most previous studies on traditional, clinic‐based spirometry, which reported decreased measurement quality in children younger than 10 years [14, 15]. Furthermore, a study focusing on children aged 5–10 years with newly diagnosed asthma found that younger children were less likely to perform reproducible tests at home with a computer‐based spirometer [16]. However, two recent studies investigating smartphone spirometry reported different findings: one study in children aged 6–16 years observed no correlation between age and average quality grade [13], while another study including both children and adults even found lower reproducibility in teenagers compared to children [12]. A possible explanation could be that the home setting allowed children to practice the manoeuvre repeatedly at their own pace and with familiar caregivers, potentially reducing anxiety and improving performance compared to a single supervised clinic visit.

Adult supervision during home measurements was common, especially in younger children, but did not significantly impact measurement quality in this study. This might reflect effective initial training, or suggest that older children are capable of reliable independent testing. However, the lack of association should be interpreted cautiously, as supervision was self‐reported and not standardised.

Social factors such as parental education and household smoking showed limited association with spirometry outcomes, indicating that these do not substantially affect home measurement feasibility in this population. The only significant associations observed were between number of siblings and measurement frequency; however, these should be interpreted with caution given the exploratory nature of the analyses and the number of comparisons performed without correction for multiple testing. The role of these and other psychosocial factors requires further investigation in larger cohorts.

Several limitations should be considered when interpreting our findings. The study was observational and non‐randomised, and there was a possible bias due to non‐blinded manual grading. The number of eligible patients who declined participation was not systematically documented, which limits the assessment of selection bias. Missing data were not imputed, and analyses were based on available data only, which may introduce bias if data were not missing at random. Supervision during home measurements was not standardised but left to the discretion of families, which could introduce variability. Our study cohort of children with suspected asthma can only be partially generalised to populations with an established asthma diagnosis or other respiratory diseases. Additionally, the relatively small proportion of fully completed 7‐day protocols indicates that longer‐term adherence remains a challenge and should be addressed in future research. Furthermore, symptom data collected via the mobile app were not available for analysis due to data integration issues, precluding an investigation of the potential influence of asthma symptoms on measurement compliance and quality. A direct comparison of the measurement quality or spirometry results with conventional clinical spirometry data was not possible, as the three participating centres used different lung function systems.

In conclusion, smartphone spirometry is a feasible method for repeated lung function assessment at home in children with suspected asthma, yielding acceptable measurement quality in a substantial proportion of attempts. Automated quality grading shows high accordance with manual grading but requires cautious interpretation to identify individual cases with deviations. However, we observed moderate compliance, underlining the importance of patient engagement and potentially, technical or motivational support to optimise home spirometry outcomes. These results support the further evaluation of home‐based smartphone spirometry in children with suspected asthma, including its potential role in the diagnostic process, while highlighting areas for improvement in measurement quality assurance and adherence.

## Author Contributions

**Antonia Knopek:** data curation, writing – original draft, conceptualisation, investigation, methodology, validation, formal analysis, visualisation, writing – review and editing. **Hanna Fabinyi:** data curation, investigation, writing – review and editing. **Viktoria Zach:** data curation, investigation, writing – review and editing. **Anna Beliveau:** data curation, writing – review and editing, investigation. **Nina Luisa Hoekstra:** investigation, writing – review and editing. **Carlotta Nüssing:** data curation, investigation, writing – review and editing, formal analysis. **Katharina Kainz:** data curation, writing – review and editing, investigation. **Swantje Weisser:** conceptualisation, methodology, writing – review and editing. **Anna Zschocke:** data curation, supervision; writing – review and editing, investigation, resources. **Angela Zacharasiewicz:** supervision, data curation, investigation, writing – review and editing, resources. **Christiane Lex:** project administration, data curation, supervision, writing – review and editing, conceptualization, investigation, funding acquisition, methodology, resources.

## Funding

The authors have nothing to report.

## Conflicts of Interest

The authors declare no conflicts of interest.

## Supporting information


Supporting File

## Data Availability

The data that support the findings of this study are available from the corresponding author upon reasonable request.
