# Supplementary material for: Feasibility and Measurement Quality of Home‐Based Smartphone Spirometry in Children With Suspected Asthma: A Multicentre, Prospective Study
Source: Pediatr Pulmonol. 2026 Aug 2;61(8):e71768. doi: 10.1002/ppul.71768 (PMC13430289; doi:10.1002/ppul.71768)
Supplement: Supplementary file 1 — Supporting File [file PPUL-61-0-s001.docx]

Supplementary Table S1: Associations between age, supervision, social factors and measurement-related parameters (Spearman-Rho analyses)

|  |  | **Number of measurement days** | **Number of measurement series** | **Number of acceptable FEV₁ attempts (manual)** | **Number of acceptable FEV₁ attempts (automated)** | **Number of acceptable FVC attempts (manual)** | **Number of acceptable FEV₁ attempts (automated)** | **Number of measurements with A-C for FEV_1_ & FVC (manual)** | **Number of measurements with A-C for FEV_1_ & FVC (automated)** |
| --- | --- | --- | --- | --- | --- | --- | --- | --- | --- |
| **Age** | Correlation coefficient | 0.010 | 0.006 | 0.075 | 0.042 | 0.029 | 0.037 | -0.049 | -0.061 |
|  | p-value  (two-tailed) | 0.917 | 0.953 | 0.454 | 0.680 | 0.774 | 0.711 | 0.638 | 0.562 |
|  | N | 101 | 101 | 101 | 101 | 101 | 101 | 94 | 94 |
| **Super- vision** | Correlation coefficient | 0.069 | 0.094 | 0.050 | 0.099 | 0.116 | 0.116 | 0.111 | 0.135 |
|  | p-value  (two-tailed) | 0.554 | 0.419 | 0.666 | 0.395 | 0.316 | 0.318 | 0.342 | 0.246 |
|  | N | 76 | 76 | 76 | 76 | 76 | 76 | 76 | 76 |
| **Parental education** | Correlation coefficient | 0.017 | -0.001 | -0.063 | -0.039 | -0.048 | -0.035 | -0.045 | -0.047 |
|  | p-value  (two-tailed) | 0.870 | 0.994 | 0.551 | 0.714 | 0.649 | 0.737 | 0.684 | 0.668 |
|  | N | 93 | 93 | 93 | 93 | 93 | 93 | 86 | 86 |
| **Number of siblings** | Correlation coefficient | **-0,208** | **-0.231** | -0.095 | -0,079 | -0.052 | -0.081 | 0.001 | 0.001 |
|  | p-value  (two-tailed) | **0.048** | **0.028** | 0.372 | 0.372 | 0.457 | 0.444 | 0.996 | 0.995 |
|  | N | **91** | **91** | 91 | 91 | 91 | 91 | 84 | 84 |
| **House-hold smoking** | Correlation coefficient | -0,079 | 0.014 | 0.037 | 0.083 | 0.070 | 0,084 | -0.036 | 0.081 |
|  | p-value  (two-tailed) | 0.484 | 0.902 | 0.743 | 0.463 | 0.534 | 0.454 | 0.756 | 0.489 |
|  | N | 81 | 81 | 81 | 81 | 81 | 81 | 76 | 76 |
|  |  |  |  |  |  |  |  |  |  |

N = number of participants with available data for the respective variable
